# Inhibiting H2AX Can Ameliorate Myocardial Ischemia/Reperfusion Injury by Regulating P53/JNK Signaling Pathway

**DOI:** 10.1155/2024/1905996

**Published:** 2024-09-03

**Authors:** Ziyang Yu, Yirong Teng, Hongbo Yang, Yudi Wang, Xichen Li, Lei Feng, Wenbo Xu, Yinglu Hao, Yanping Li

**Affiliations:** ^1^ Department of Cardiology The 6th Affiliated Hospital of Kunming Medical University The People's Hospital of Yuxi City, Yuxi, Yunnan, China; ^2^ Department of General Practice The 6th Affiliated Hospital of Kunming Medical University The People's Hospital of Yuxi City, Yuxi, Yunnan, China; ^3^ Department of Cardiology Fuwai Yunnan Hospital Chinese Academy of Medical Sciences, Kunming, Yunnan, China; ^4^ Department of Laboratory Yan'an Hospital of Kunming City, Kunming, Yunnan, China; ^5^ Department of Laboratory The 6th Affiliated Hospital of Kunming Medical University The People's Hospital of Yuxi City, Yuxi, Yunnan, China

## Abstract

Myocardial ischemia-reperfusion (I/R) injury is a significant area of focus in cardiovascular disease research. I/R injury can increase intracellular oxidative stress, leading to DNA damage. H2AX plays a crucial role in DNA repair. This study utilized mouse and cell models of myocardial I/R to investigate the impact of H2AX on cardiomyocytes during I/R. This study initially assessed the expression of H2AX in MI/R mice compared to a sham surgery group. Subsequently, cardiac function, infarct area, and mitochondrial damage were evaluated after inhibiting H2AX in MI/R mice and a negative control group. Furthermore, the study delved into the molecular mechanisms by analyzing the expression of H2AX, P53, p-JNK, SHP2, p-SHP2, p-RAS, parkin, Drp1, Cyt-C, Caspase-3, and Caspase-8 in cardiomyocytes following the addition of JNK or P53 agonists. The results from western blotting in vivo indicated significantly higher H2AX expression in the MI/R group compared to the sham group. Inhibiting H2AX improved cardiac function, reduced myocardial infarct area, and mitigated mitochondrial damage in the MI/R group. In vitro experiments demonstrated that inhibiting H2AX could attenuate mitochondrial damage and apoptosis in myocardial cells by modulating the P53 and JNK signaling pathways. These findings suggested that inhibiting H2AX may alleviate myocardial I/R injury through the regulation of the P53/JNK pathway, highlighting H2AX as a potential target for the treatment of myocardial ischemia/reperfusion injury.

## 1. Introduction

With the economic surge propelling an upturn in living conditions, a parallel rise in health complications, notably age-associated ailments like cardiovascular disorders, has been observed [1]. Epidemiological data indicate a relentless climb in the prevalence of cardiovascular maladies. Coronary artery disease, primarily characterized by myocardial ischemia-reperfusion (I/R) injury, stands as a predominant contributor to global mortality and morbidity [2]. The paradoxical exacerbation of cardiac damage post-restoration of blood flow subsequent to ischemic episodes defines myocardial I/R injury. This condition precipitates profound cardiac dysfunction and can culminate in myocardial infarction, a significant factor in cardiovascular mortality. The quest for efficacious preventative and therapeutic strategies for I/R injury is a pressing concern within the medical research community [3, 4]. Addressing the cardiac detriments and functional impairments induced by I/R is thus of paramount importance [5, 6].

In the milieu of myocardial ischemia, the deprivation of oxygen thwarts the standard metabolic process of oxidative phosphorylation within myocardial cells. This oxygen scarcity leads to an overabundance of hydrogen ions, a dip in intracellular pH, a surge in calcium ion concentration, and an intensified oxidative stress response [7–9]. Reperfusion, despite reinstating oxygenated blood flow, paradoxically intensifies oxidative stress within the myocardium. Such stress instigates lipid peroxidation in cell membranes, protein oxidation, DNA damage, and cytochrome C release, inflicting harm on myocardial and vascular endothelial cells [7–9]. Notably, it triggers apoptotic and inflammatory pathways, further compromising cardiac integrity [7–9]. Furthermore, oxidative stress not only leads to direct damage to cellular structure and function but also activates the P53/JNK signaling pathway, which is a key pathway for cells to respond to DNA damage and initiate programmed cell death [10]. The discovery underscores the pivotal influence of the P53/JNK cascade in modulating apoptosis and inflammatory reactions subsequent to cardiac ischemia-reperfusion damage. It also sheds fresh light on the involvement of H2AX within this context.

Within myocardial ischemic conditions, H2AX, an isoform of histone H2A, is instrumental in mending DNA double-helix ruptures [11]. The phosphorylated state of H2AX serves as a beacon for genomic instability, orchestrating the assembly of DNA repair complexes and bolstering the cellular mechanisms for DNA damage detection and amendment [12]. H2AX's role in myocardial ischemia-reperfusion (I/R) is complex and layered. Moreover, the presence of phosphorylated H2AX has been implicated in triggering apoptotic pathways, culminating in the systematic elimination of impaired myocardial cells [13]. This indicates a modulatory function for H2AX in the apoptotic circuits engaged during myocardial I/R incidents [14–17].

H2AX exerts a modulatory effect on the apoptotic processes initiated during myocardial I/R episodes, indicating its potential pivotal role in the orchestrated demise of cardiac muscle cells. Reflecting on these essential functions of H2AX in myocardial I/R trauma, this investigation developed a murine model of myocardial I/R and conducted a sequence of in vivo and ex vivo analyses to delineate the impact of H2AX on myocardial I/R and to elucidate its underlying molecular dynamics.

## 2. Materials and Methods

### 2.1. Experimental Animals and Grouping

In this study, a cohort of thirty-six adult male C57BL/6 mice, procured from Henan Skobes Biotechnology Co., Ltd, Henan, China, each weighing an average of 25 ± 2 g, were acclimatized to a controlled environment with a 12-hour light/dark cycle at a stable temperature of 25°C. They had unrestricted access to standard chow and water. The subjects were stratified into six distinct cohorts, each comprising six mice: the sham-operated group, the myocardial ischemia-reperfusion (MI/R) group, the sham-negative control (sham-NC)/reperfusion-negative control (R-NC) group, and the MI/R group treated with an H2AX inhibitor.

### 2.2. Mouse Model of Myocardial Ischemia/Reperfusion (MI/R)

For the experimental procedure, the mice received an intraperitoneal dose of 1% sodium pentobarbital (50 mg/kg, sourced from Sigma, St. Louis, USA) to induce anesthesia. Subsequent to tracheal intubation, the subjects were connected to a rodent ventilator (model ALC-V8, Shanghai, China) for mechanical respiration. Sterilization of the thoracic surface preceded a left-sided thoracotomy between the fourth and fifth ribs to reveal the cardiac structure. Ischemic conditions were simulated by the occlusion of the left anterior descending (LAD) coronary artery using a surgical suture for a duration of 30 minutes, succeeded by a 180 minute reperfusion phase initiated by suture release. The onset of ischemia was visually confirmed by a discernible discoloration in the myocardial tissue and the designated risk zone. Reperfusion was verified upon the removal of the occlusion and closure of the surgical site. In both the sham-operated group treated with H2AX inhibitor and the MI/R cohort receiving the same inhibitor, the compound was directly administered into the cardiac muscle tissue.

### 2.3. ELISA

Antigens for lactate dehydrogenase (LDH), aspartate aminotransferase (AST), and creatine kinase (CK) were immobilized onto a microtiter plate, each well receiving 100 *μ*L, and incubated at 37°C for a span of 4 hours. Following incubation, the wells were emptied. A 5% solution of fetal bovine serum was then introduced into each well, serving as a blocking agent against nonspecific interactions over a 40 minute incubation at 37°C. Subsequent to a triad of washes, the wells were filled with 100 *μ*L of the appropriately diluted samples and incubated once more at 37°C for 40 to 60 minutes. After three additional washes, wells were treated with enzyme-conjugated antibodies and incubated for 60 minutes at 37°C. A final series of three washes preceded the addition of TMB substrate, ceasing the enzymatic reaction. The optical density (OD) of each well was quantified at a 450 nm wavelength using an ELISA microplate reader. The concentrations of LDH, AST, and CK were deduced by plotting the OD values against a pre-established standard curve.

### 2.4. 2,3,5-Triphenyltetrazolium Chloride (TTC) Staining

The cardiac specimens from each experimental subset underwent a perfusion process with 3 mL of 2% Evans blue dye (procured from Thermo Fisher) administered via the aortic conduit. Subsequently, they were methodically arranged on an icy metal surface and subjected to a −20°C environment for an overnight period. Post-freezing, the hearts were sectioned into 2 mm coronal segments utilizing a chilled, keen-edged instrument, in alignment with the longitudinal axis of the left ventricular chamber. These sections were then immersed in a 2% triphenyltetrazolium chloride (TTC) solution (sourced from Sigma Aldrich) maintained at 37°C for a quarter-hour, followed by a phosphate-buffered saline (PBS) rinse, and ultimately preserved in a 4% formalin solution.

### 2.5. Mouse Echocardiography for Cardiac Function Assessment

Upon the completion of the modeling phase and a fortnight of pharmacological intervention, the murine subjects were rendered unconscious using isoflurane. Subsequent to depilation of the thoracic region, the post-therapeutic cardiac architecture was scrutinized utilizing the Vevo 2100 imaging apparatus. Bidimensional echocardiographic snapshots were captured, delineating structural cardiac parameters such as left ventricular end-diastolic diameter (LVID; d), left ventricular end-systolic diameter (LVID; s), systolic thickness of the left ventricular anterior wall (LVAW; s), and diastolic thickness of the left ventricular anterior wall (LVAW; d). Functional cardiac indices were represented by the ejection fraction (EF) and fractional shortening (FS).

### 2.6. Masson's Staining

Post-dissection, the cardiac tissues of the mice were preserved in 4% paraformaldehyde, subsequently encased within paraffin, and sectioned into 4 *μ*m slices. These slices underwent a staining regimen with specific binding agents. Following a cleansing cycle, the sections were treated with Sirius Red solution (courtesy of Thermo Fisher) for a 3 minute duration, subjected to a differentiation process using a hydrochloric acid-alcohol solution, and then stained with Ponceau acid fuchsin for 10 minutes. This was succeeded by a 4 minute differentiation stage in 1% phosphomolybdic acid solution. The sections were further stained with a light green solution for 5 minutes and set in a neutral resin medium. The final step involved capturing the stained sections' images through microscopic photography.

### 2.7. Transmission Electron Microscopy (TEM)

The specimens obtained were sectioned into trifurcated segments and subjected to stabilization using a 2.5% glutaraldehyde solution. Subsequently, they underwent a secondary fixation with a 1% osmium tetroxide preparation, followed by encapsulation within an epoxy resin matrix for an extended period. Post-embedding, the segments were sequentially treated with uranyl acetate for a duration of half an hour and lead citrate for a brief five-minute interval. The prepared samples were then examined utilizing a Philips CM120 transmission electron microscope to assess ultrastructural details.

### 2.8. Cell Treatment and Grouping

The HL-1 murine cardiomyocyte strain was procured from Procell Life Science&Technology Co.,Ltd.(Wuhan, China). These cells were propagated in MEM medium, enriched with 10% fetal bovine serum and 1% double antibody. The HL-1 culture was subjected to a pre-equilibration phase with a hypoxic gas mixture (95% nitrogen and 5% oxygen) for a minimum of two hours. This was succeeded by a four-hour hypoxic exposure within an incubator set to 95% nitrogen and 5% carbon dioxide at a controlled temperature of 37°C. Following this, the culture medium was refreshed, and the cells underwent a reoxygenation process in an atmosphere containing 95% air and 5% carbon dioxide for two hours. To delineate the negative control (NC) and the H2AX inhibitor-treated groups, HL-1 cells were administered an H2AX inhibitor. Furthermore, the cardiomyocytes within both the NC and H2AX inhibitor cohorts received treatment with the JNK pathway stimulant anisomycin and the P53 pathway activator MeOIstPyrd.

### 2.9. Western Blotting

Protein samples were isolated utilizing a dedicated extraction kit, and concentrations were quantified. A measure of 50 *µ*g of the isolated protein underwent electrophoretic separation via SDS-PAGE, followed by the transference of the resolved proteins onto a nitrocellulose membrane through the blotting technique. To prevent nonspecific interactions, the membrane was saturated with 3.0% skim milk, then incubated with a suite of primary antibodies targeting H2AX (Abcam, ab26350, 1 : 1000), P53 (Abcam, ab307802, 1 : 1000), p-JNK (Abcam, ab32509, 1 : 1000), SHP2 (Abcam, ab62322, 1 : 1000), p-SHP2 (Abcam, ab32509, 1 : 1000), p-RAS (Cell Signaling, #3321, 1 : 1000), parkin (Abcam, ab77924, 1 : 2000), Drp1 (Abcam, ab184247, 1 : 1000), Cyt-C (Abcam, ab133504, 1 : 5000), Caspase-3 (Abcam, ab32351, 1 : 5000), and Caspase-8 (Abcam, ab32397, 1 : 1000), as well as GAPDH (Abcam, ab9485, 1 : 2500) at 4°C. Subsequent to thorough rinsing, the membrane was exposed to horseradish peroxidase-tagged goat anti-rabbit secondary antibody (Abcam, ab97080, 1 : 5000) at ambient temperature for a span of two hours. The final step involved capturing chemiluminescent signals and quantifying grayscale intensities via analytical software.

### 2.10. Statistical Analysis

The statistical evaluation and data manipulation were executed utilizing GraphPad Prism version 9.0. The quantitative data are articulated as mean ± standard deviation. For the assessment of differences across multiple cohorts, the chi-square test was employed, whereas the independent sample *t*-test facilitated the comparative analysis between two distinct groups. A *p* value falling below the threshold of 0.05 was deemed to denote statistical significance.

## 3. Results

### 3.1. The Expression of p-H2AX in the Myocardial Tissue of Mice Subjected to Myocardial Ischemia-Reperfusion Significantly Increased

**Figure 1:**
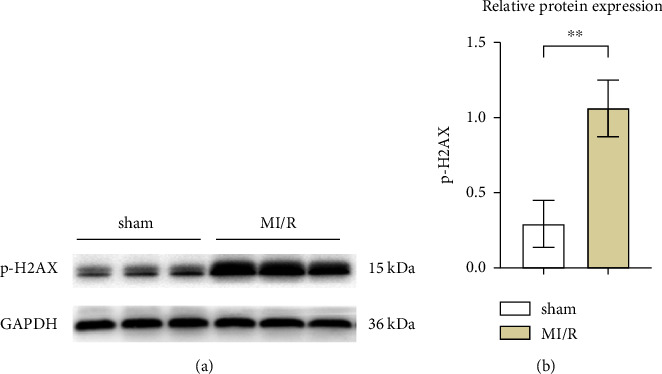
Increased expression of p-H2AX in myocardial tissue of myocardial ischemia/reperfusion mice. (a) Western blotting analysis of p-H2AX in sham group and MI/R group mice. (b) Relative expression of p-H2AX in sham group and MI/R group mice. ^*∗∗*^*P* < 0.001.

Initially, the study aimed to elucidate the function of phosphorylated H2AX in myocardial ischemia-reperfusion (I/R) events in murine models. To this end, a myocardial I/R model was established alongside a control cohort undergoing sham operations. Western blot analyses revealed a marked upregulation in the expression levels of phosphorylated H2AX in the myocardial I/R group when contrasted with the sham-operated counterparts, as depicted in Figures 1(a) and 1(b).

### 3.2. Inhibition of H2AX Can Improve Cardiac Function in Mice Subjected to Myocardial Ischemia-Reperfusion

**Figure 2:**
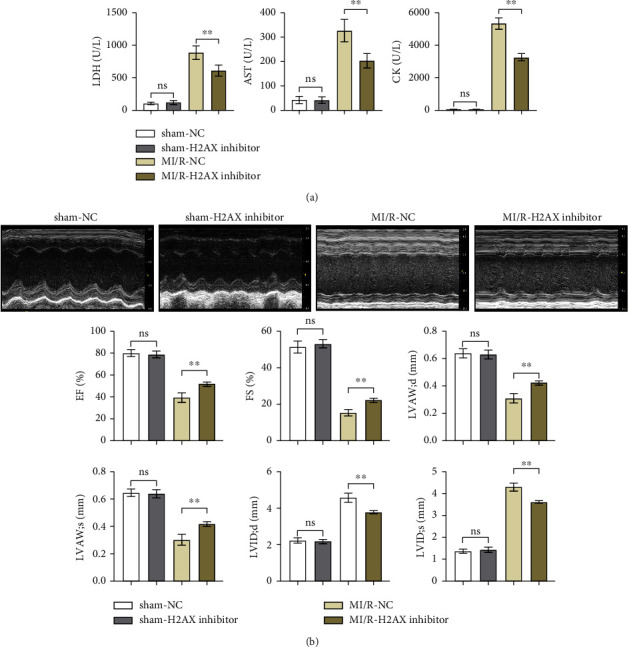
Inhibiting H2AX can reduce heart function in myocardial ischemia/reperfusion mice. (a) Concentrations of CK, LDH, and AST in mouse serum in sham group and MI/R group with or without H2AX inhibitor, determined by ELISA. (b) Ultrasound images of mice and statistics of EF, FS, LVAW; d, LVAW; s, LVID; d, and LVID; s in sham group and MI/R group with or without H2AX inhibitor. ^*∗∗*^*P* < 0.001.

Lactate dehydrogenase (LDH), a cytoplasmic enzyme, is pivotal in glycolysis, catalyzing the interconversion of lactate and pyruvate. Aspartate aminotransferase (AST), ubiquitous in cardiac, hepatic, muscular, and renal cells, serves as a biomarker for cardiac afflictions when assayed in serum. Creatine kinase (CK), integral to muscle function and energy homeostasis, is indicative of myocardial injury when elevated in the bloodstream. ELISA data indicated that LDH, AST, and CK levels in both sham-operated negative control (sham-NC) and sham-H2AX inhibitor groups remained low without notable variance. Contrastingly, these enzyme concentrations were elevated in the myocardial ischemia/reperfusion negative control (MI/R-NC) and MI/R-H2AX inhibitor groups, as shown in Figure 2(a). Notably, the MI/R-H2AX inhibitor group exhibited a substantial reduction in these enzymes compared to the MI/R-NC group, suggesting a mitigative effect of H2AX inhibition on enzymatic release post-injury.

Cardiac ultrasonographic evaluation of the mice revealed no significant disparities in EF, FS, LVAW; d, and LVAW; *s*, as well as LVID; d and LVID; s between the sham-NC and sham-H2AX inhibitor groups. However, in the MI/R-H2AX inhibitor group, there was a notable augmentation in EF, FS, LVAW; d, and LVAW; s, alongside a reduction in LVID; d and LVID; s when juxtaposed with the MI/R-NC group. These findings, depicted in Figure 2(b), underscore the potential of H2AX inhibition in enhancing myocardial function following I/R injury in mice.

### 3.3. Inhibition of H2AX Reduces Myocardial Infarct Size and Mitochondrial Injury in Myocardial Ischemia-Reperfusion Mice

**Figure 3:**
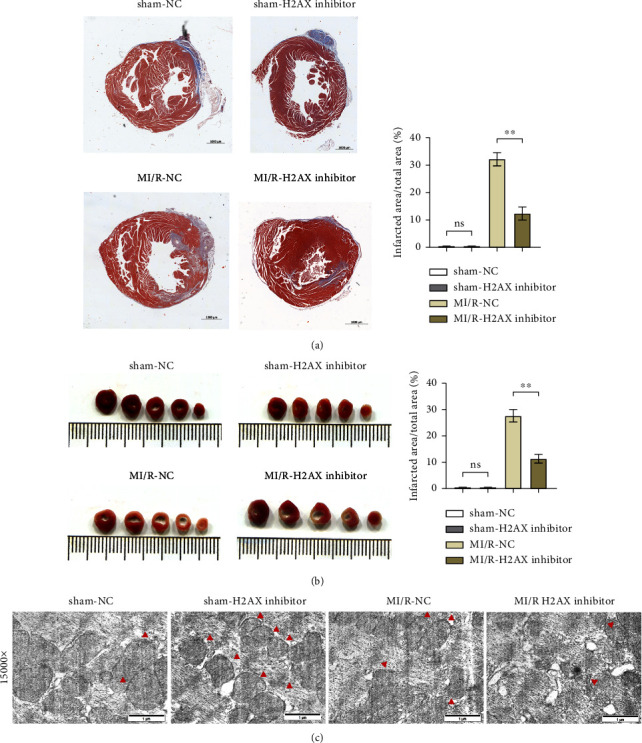
Inhibiting H2AX can reduce the infarct area in myocardial ischemia/reperfusion mice. (a) Masson staining and infarct area ratio in sham group and MI/R group with or without H2AX inhibitor. (b) TTC staining results and infarct area ratio in sham group and MI/R group with or without H2AX inhibitor (*n* = 5). (c) Inhibiting H2AX can reduce mitochondrial damage in myocardial ischemia/reperfusion mice. ^*∗∗*^*P* < 0.001.

Masson's trichrome staining and TTC assay elucidated the myocardial infarction extent, revealing minimal infarct sizes in both the sham-NC and sham-H2AX inhibitor groups, with no discernible differences. Conversely, the myocardial infarction proportions were notably higher in the MI/R-NC and MI/R-H2AX inhibitor groups. A significant diminution in infarct size was observed in the MI/R-H2AX inhibitor group when juxtaposed with the MI/R-NC group, as illustrated in Figure 3(a) and 3(b). Further, the impact of H2AX inhibition on mitochondrial integrity was assessed in the myocardial I/R model via transmission electron microscopy, which offers detailed visualization of cellular architecture. In the sham cohorts, mitochondrial morphology remained intact. However, in the MI/R-NC group, mitochondria exhibited pronounced swelling and structural aberrations, including increased vacuolation, disrupted cristae, and compromised outer membranes. In contrast, the MI/R-H2AX inhibitor group displayed mitigated mitochondrial swelling and cristae deformation, with infrequent occurrences of membrane disruption, as shown in Figure 3(c). These observations suggest that H2AX inhibition may confer protection against mitochondrial damage in myocardial ischemia-reperfusion scenarios.

### 3.4. Inhibition of H2AX Can Suppress Mitochondrial Damage and the Apoptosis of Myocardial Cells by Regulating P53 and JNK Signaling Pathway

To delve deeper into the modulation of I/R injury by H2AX, we conducted western blot assays for p-H2AX, P53, p-JNK, SHP, p-SHP2, p-RAS, parkin, Drp1, Cyt-C, Caspase-3, and Caspase-8. Research indicates that augmented ROS levels may instigate the association of P53 with the SHP2 promoter, leading to a suppression of Src homology 2 domain-containing phosphatase-2 (SHP2) expression and a marginal reduction in P53 levels. RAS, a member of the small *G* protein family, orchestrates a variety of cellular signaling cascades, including those governing cell viability, proliferation, and differentiation. SHP2's dephosphorylation of RAS-GTPase activating protein (RAS-GAP) attenuates its inhibitory effect on RAS, thereby potentiating RAS signaling and frequently culminating in the attenuation of P53 activity [18, 19]. JNK, or c-Jun N-terminal kinase, is a crucial kinase implicated in cellular stress responses [20], and it is postulated that H2AX may facilitate the phosphorylation of JNK [20]. Parkin is instrumental in mitochondrial maintenance, influencing cellular survival and metabolic equilibrium by modulating the interplay between mitochondria and apoptotic processes [21, 22]. Drp1, a mitochondrial fission regulator, dynamically shapes mitochondrial morphology. Cytochrome C (Cyt-C), a vital mitochondrial protein, is central to both the respiratory chain and the apoptotic pathway [23–25]. Additionally, Caspase-3 and Caspase-8 are pivotal in the execution phase of intrinsic apoptosis.

**Figure 4:**
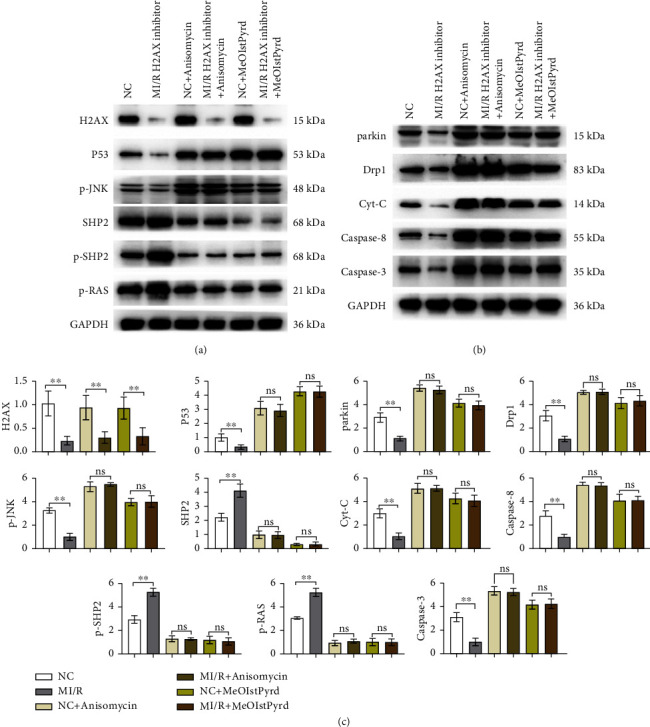
Inhibiting H2AX can suppress the expression of p-JNK and mitochondrial autophagy and fusion, as well as attenuate apoptosis in cardiac myocytes through the P53/SHP2 signaling pathway. (a) Western blotting and relative expression of p-H2AX, P53, p-JNK, SHP, p-SHP2, and p-RAS. (b) Western blotting and relative expression of parkin, Drp1, Cyt-C, Caspase-3, and Caspase-8. ^*∗∗*^*P* < 0.001. (c) Western blot grayscale statistics.

Through western blot analysis, it was observed that the suppression of H2AX led to a notable decrease in the levels of phosphorylated H2AX, P53, phosphorylated JNK, parkin, Drp1, cytochrome c, Caspase-3, and Caspase-8 within the H2AX inhibitor cohort relative to the normal control (NC) group. Conversely, the levels of SHP2, phosphorylated SHP2, and phosphorylated RAS were markedly elevated. Subsequent administration of JNK or P53 activators did not alter the levels of phosphorylated H2AX in either the NC or H2AX inhibitor groups; however, the expression of P53, phosphorylated JNK, parkin, Drp1, cytochrome c, Caspase-3, and Caspase-8 was augmented, while SHP2, phosphorylated SHP2, and phosphorylated RAS were diminished, with no significant disparities observed. Thus, the inhibition of H2AX is implicated in mitigating mitochondrial impairment and cardiomyocyte apoptosis through modulation of the P53 and JNK signaling cascades (Figure 4).

## 4. Discussion

Cardiovascular ailments stand as a prominent subset of public health concerns, presenting a grave risk to human physiological well-being. The issue of myocardial I/R injury is increasingly coming to the fore. Such injuries trigger a surge in reactive oxygen species (ROS), thereby aggravating cardiac harm [5, 26]. Cellular exposure to oxidative stress can lead to both direct and indirect oxidative degradation of DNA, manifesting in double-stranded DNA breaks. These breaks prompt a rapid phosphorylation of the H2AX protein, which in turn fosters the formation of *γ*-H2AX complexes aimed at repairing the damaged DNA [27–29]. In the current investigation, initial observations revealed a pronounced elevation in the relative protein expression of p-H2AX in the MI/R murine model. Inhibition of H2AX was associated with enhanced cardiac functionality, diminution of the infarct region, and mitigation of mitochondrial detriment in myocardial ischemia/reperfusion models in mice. These findings indicate that H2AX inhibition may indeed mitigate myocardial ischemia/reperfusion injury. In light of this, our study probes into the involvement of H2AX in the context of myocardial ischemia/reperfusion incidents.

**Figure 5:**
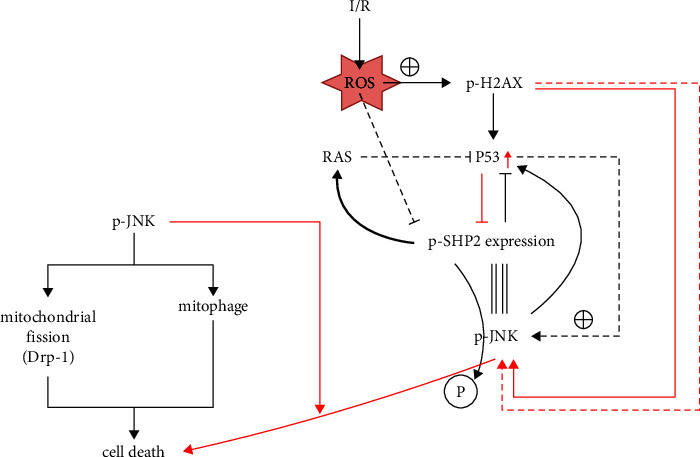
H2AX mediates myocardial ischemia/reperfusion injury by regulating P53 and JNK signaling pathways.

The present investigation reveals that cardiac tissue-specific delivery of H2AX inhibitors can be achieved via targeted delivery mechanisms, including nanoparticle-based systems or antibody-drug conjugates [30]. Such methodologies could herald a novel therapeutic paradigm for cardiac ailments, particularly where meticulous drug targeting and minimization of systemic adverse reactions are imperative, as shown in Figure 5.

In addition to triggering a surge in ROS, multiple signaling pathways are activated in I/R injury, including the JNK pathway [31]. JNK is a stress-activated protein kinase that is responsive to cellular responses to a variety of stress signals, such as heat shock, ischemia, osmotic changes, and inflammatory factors [32]. JNK is a protein kinase that is activated in the heart, brain, and brain. Studies have shown that JNK-mediated signaling pathways play a crucial role in cardiac and cerebral ischemia/reperfusion injury, and that JNK-related mechanisms are involved in cardiac and cerebral preconditioning and postconditioning [33, 34]. P53, a pivotal guardian of the cellular genome, orchestrates a critical response to stressors, particularly DNA damage [35]. Upon activation, P53 modulates a plethora of target genes, encompassing those involved in DNA repair and apoptosis, thereby safeguarding genomic integrity and countering DNA damage [36]. Subsequent inquiries delved into the molecular underpinnings. Western blot analyses indicated a substantial reduction in the relative expression of p-H2AX, P53, and p-JNK, concomitant with an upsurge in SHP, p-SHP2, and RAS levels following H2AX inhibition. The introduction of JNK or P53 agonists did not modify the relative expression of p-H2AX in either the NC or H2AX inhibitor groups. However, there was a notable increase in the relative expression of P53 and p-JNK, and the previously observed significant disparities between the NC and H2AX inhibitor groups were no longer present. The potential of JNK inhibitors in the clinical treatment of I/R has attracted the attention of researchers. Although the JNK pathway has roles in a variety of physiologic and pathologic conditions, the development of JNK inhibitors may provide new strategies for the treatment of I/R injury [37]. For example, the JNK inhibitor SP600125 has shown potential for protective effects against I/R injury in animal models [38]. However, due to the multifunctional nature of the JNK pathway, direct inhibition of JNK may have detrimental consequences and thus requires caution in clinical application. In summary, although JNK inhibitors have the potential to mimic the effects of H2AX inhibitors, additional safety and efficacy studies are needed before they can be used in clinical therapy.

The inhibition of the DNA damage response (DDR) pathway can precipitate a host of adverse cellular and tissue reactions, including cellular aging, apoptosis, and inflammation—key contributors to the development of cardiovascular diseases [39]. The issue of cardiotoxicity at later stages is particularly problematic in the realm of cancer therapy-related toxicity, with DNA damage-induced cardiac inflammation potentially resulting in heart failure or mortality. In our study, we diligently monitored for any toxicological indications within cardiac or other tissues. While our observations did not reveal substantial cardiotoxicity, the prolonged effects of DDR inhibition and its broader tissue implications warrant attention. Hence, we propose continuous cardiac monitoring and scrutiny of additional tissues that might be implicated in subsequent clinical investigations involving H2AX inhibitors.

## 5. Conclusion

Our findings indicate a marked upregulation of p-H2AX in the myocardial tissues of mice undergoing myocardial ischemia-reperfusion. The attenuation of H2AX was correlated with improvements in cardiac performance, a reduction in the myocardial infarct size, and lessened mitochondrial injury in the I/R model. Moreover, the modulation of H2AX activity was found to diminish mitochondrial impairment and cardiomyocyte apoptosis, which is mediated through the P53 and JNK signaling pathways. Collectively, these observations suggest that targeting H2AX could be a viable therapeutic strategy for mitigating myocardial ischemia/reperfusion injuries.

## Data Availability

Data availability is not applicable to this article as no new data were created or analyzed in this study.
